# Mediastinal angiosarcoma mimicking constrictive pericarditis and aortic dissection: a case report

**DOI:** 10.1186/s44215-024-00147-5

**Published:** 2024-02-22

**Authors:** Koki Ikemoto, Satoshi Numata, Takuma Kobayashi, Hitoshi Yaku

**Affiliations:** Department of Cardiovascular Surgery, Kyoto Prefectural University of Medicine, 465 Kajii-cho, Kawaramachi-Hirokoji, Kamigyo-ku, Kyoto, 602-8566 Japan

**Keywords:** Mediastinal angiosarcoma, Constrictive pericarditis, Aortic dissection, Coronary artery disease, Hyaluronic acid

## Abstract

**Background:**

Angiosarcomas are soft-tissue sarcomas of endothelial cells that can arise from any site in the body. Primary mediastinal angiosarcoma is rare, with an extremely poor prognosis and currently no established treatment. Mediastinal angiosarcoma is often detected as a tumor mass in the heart, lung, aorta, or pulmonary artery. However, in cases where no tumor mass is found, a definitive diagnosis is difficult without tissue biopsy, as the condition shows various, non-specific clinical findings.

**Case presentation:**

A 49-year-old man had an episode of syncope while walking. Cardiac catheterization and computed tomography suggested constrictive pericarditis, aortic dissection, and coronary artery disease. Scheduled total arch replacement and coronary artery bypass grafting could not be completed because intraoperative findings indicated the presence of a malignant tumor. Only pericardiectomy was performed. Hyaluronic acid concentration in the pleural fluid was high. The diagnosis of mediastinal angiosarcoma was confirmed postoperatively.

**Conclusions:**

Mediastinal angiosarcoma might mimic multiple diseases within a single case. Hyaluronic acid concentration in the pleural fluid may be a useful indicator for mediastinal angiosarcoma diagnosis.

**Supplementary Information:**

The online version contains supplementary material available at 10.1186/s44215-024-00147-5.

## Background

Angiosarcomas are soft-tissue sarcomas of endothelial cells that can originate from anywhere in the body [1]. Primary mediastinal angiosarcoma (MA) is exceedingly rare, with an extremely poor prognosis and currently no established treatment [2]. MA is often detected as a tumor mass in a mediastinal organ such as the heart, lung, aorta, or pulmonary artery by general imaging modalities. However, especially in cases where no tumor mass is found, a definitive diagnosis of MA is difficult without tissue biopsy because the condition shows various, non-specific clinical findings caused by invasion or dissemination to adjacent organs [3, 4]. We report a case of MA that mimicked constrictive pericarditis, aortic dissection, and coronary artery disease and showed a high concentration of hyaluronic acid (HA) in the pleural effusion.

## Case presentation

**Fig. 1:**
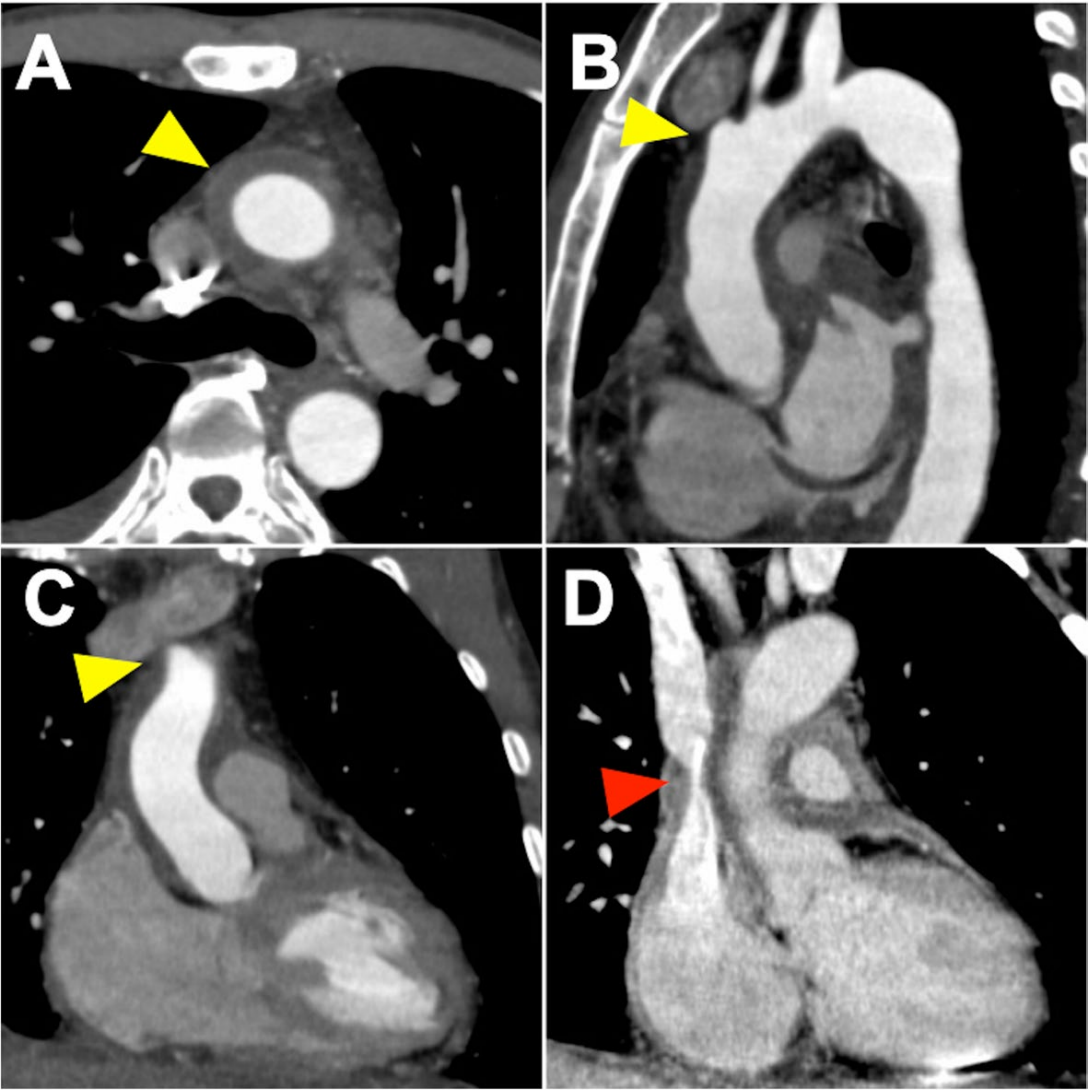
Preoperative enhanced computed tomography. The thrombosed dissection-like finding was spread from the ascending aorta (**A** yellow arrow) to the proximal aortic arch and the orifice of the right subclavian artery (**B**, **C** yellow arrows). The superior vena cava was severely shrunken next to the ascending aorta (**D** red arrow)

**Fig. 2:**
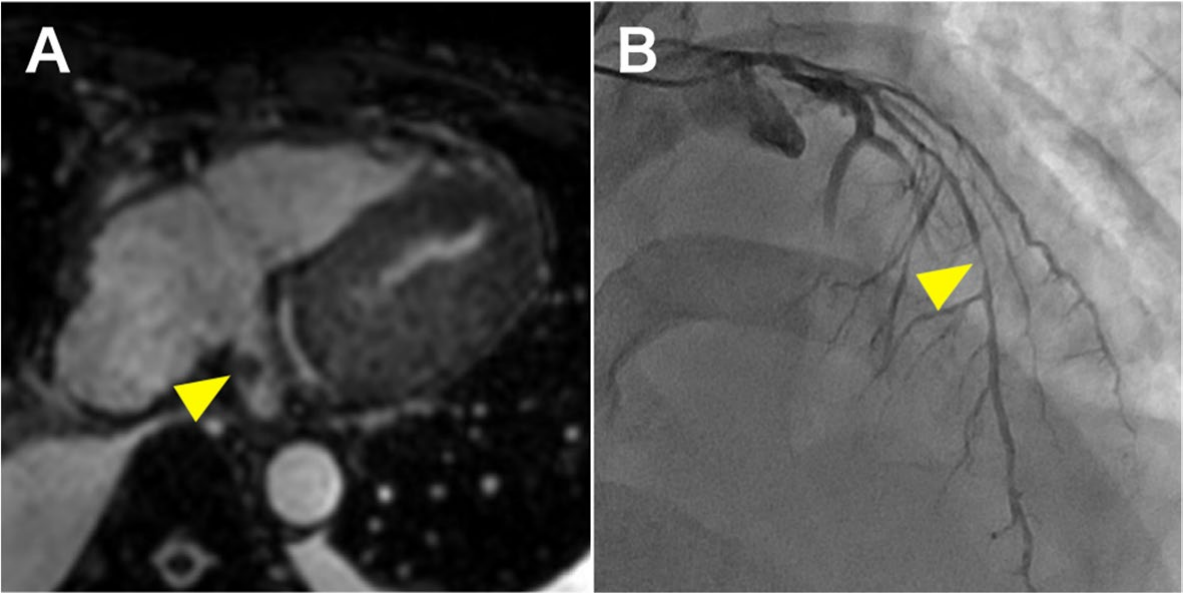
Preoperative magnetic resonance imaging and coronary angiography. A small nodule, suggesting a thrombus, was detected at the coronary sinus (**A** yellow arrow). There was significant stenosis in the middle of the left anterior descending artery (**B** yellow arrow)

A 49-year-old man, who was admitted to another hospital with neck pain, back pain, and general fatigue 2 months prior, had an episode of syncope while walking. He was diagnosed with cardiac tamponade by transthoracic echocardiography, and pericardiocentesis demonstrated bloody pericardial effusion. No malignant findings were identified by cytology. He was referred to our institution for further treatment. Enhanced computed tomography suggested intramural hematoma from the ascending aorta to the proximal aortic arch (Fig. 1A, B) and the orifice of the right subclavian artery (Fig. 1B, C), and stenosis of the superior vena cava (Fig. 1D). Magnetic resonance imaging (MRI) revealed a small nodule in the coronary sinus, suggesting a thrombus (Fig. 2A). There were no findings suggestive of malignancy (Supplementary Video 1). Cardiac catheterization showed a dip-and-plateau pattern of right ventricular pressure. There was significant stenosis of the left anterior descending artery (Fig. 2B). Transthoracic echocardiography demonstrated a small pericardial effusion and an ejection fraction of 45%, with hypokinesis from the lateral to the inferoposterior wall of the left ventricle (Supplementary Video 2). There were no tumor mass findings. Pericardiectomy, total arch replacement, and coronary artery bypass grafting were planned for the treatment of constrictive pericarditis, subacute thrombosed aortic dissection, and single-vessel coronary artery disease, respectively.

After median sternotomy, the pericardium was carefully detached from the heart (Supplementary Video 3). The epicardium could not be resected because it was firmly adherent to the epicardial myocardium. Aortic dissection was not detected. The superior vena cava was highly adherent to the ascending aorta and was shrunken as if it had been invaded by a malignant tumor. Malignant disease was highly suspected by frozen section examination of the partially resected pericardium; therefore, only pericardiectomy was performed under cardiopulmonary bypass, considering surgical invasion and patient prognosis. The right and left pericardial flaps were excised approximately 1 cm in front of the right and left phrenic nerves, respectively. A small nodule approximately 2 cm in size was detected at the coronary sinus, which was hard and involved the coronary sinus valve. There was a substantial invasion of the heart tissue, precluding safe resection. Cardiopulmonary bypass was carefully weaned off using inotropic agents. The patient was extubated on postoperative day (POD) 7, and inotropic agents were completely removed on POD 14. However, on POD 21, his heart suddenly underwent arrest while walking. Electrocardiography showed no signal from his heart, which did not react to any resuscitation effort. Peripheral veno-arterial extracorporeal oxygenation was thus subsequently induced. Although myocardial infarction was suspected, cardiac enzyme levels were not elevated after the event. The patient died 2 days later, despite undergoing intensive treatment.

**Fig. 3:**
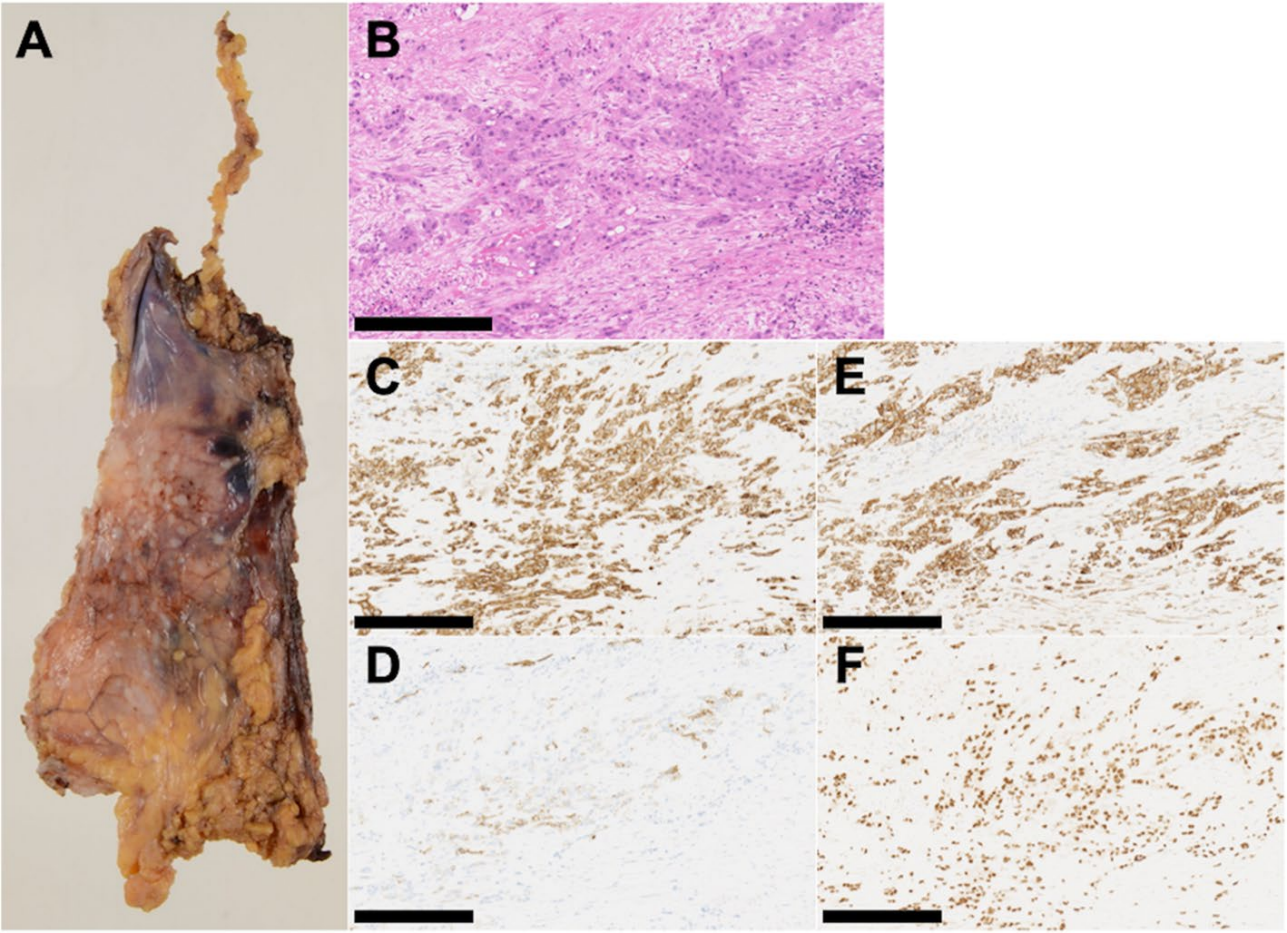
Intraoperative partially excised pericardium and immunohistochemical staining. There were multiple disseminated nodules on the pleural surface of the pericardium excised intraoperatively (**A**). Hematoxylin and eosin staining demonstrated atypical epithelioid cells proliferating in a sinusoidal pattern and vascular spaces lined by spindle cells (**B**). Immunostains were positive for CD 31, CD 34, D2-40, and ERG (**C**–**F**). Scale bar = 250 μm

Multiple disseminated nodules were detected on the pleural surface of the pericardium excised intraoperatively (Fig. 3A). Pathological analysis revealed atypical epithelioid cells proliferating in a sinusoidal pattern and vascular spaces lined by spindle cells (Fig. 3B). Immunostains were positive for CD 31, CD 34, D2-40, and ERG (Fig. 3C–F). Cytology of the pleural effusion showed atypical cells, which were closely similar to those in the pericardium. The concentration of HA in the pleural effusion was 14,500 ng/mL. The diagnosis of MA was confirmed based on these findings.

## Discussion and conclusions

MA can plausibly mimic many types of disease within a single case. MA may originate from the heart, lung, or giant vessels and is often detected as a tumor mass by echocardiography, computed tomography, and MRI. A few reports have shown angiosarcoma arising from the lung or heart to be reminiscent of a different disease, such as alveolar hemorrhage, constrictive pericarditis, intramural hematoma, and coronary insufficiency [4–8]; however, it has never been reported to mimic multiple diseases within a single case. In our reported case, the patient was diagnosed with constrictive pericarditis, subacute thrombosed aortic dissection, and single-vessel coronary artery stenosis, based on bloody pericardial effusion, neck and back pain, and clinical examination results. Moreover, the absence of an obvious tumor mass supported this diagnosis. However, all these findings were eventually shown to be caused by the MA infiltrating adjacent tissue and bleeding in the pericardium. Had we suspected the presence of a malignant tumor and performed additional examinations, the patient could have been proposed a different treatment option with a meaningful life expectancy.

MA presents various clinical and laboratory findings, such as fever, syncope, chest pain, fatigue, or cardiac tamponade, depending on the site of occurrence, metastasis, or infiltration. However, these findings are non-specific and are not strong determinants for definitive MA diagnosis. HA concentration in the pleural fluid is known to be a useful indicator for malignant mesothelioma diagnosis; however, likewise, HA levels increase in patients with other malignant tumors or benign inflammatory disease [9]. Positron emission tomography, which is often performed to detect a malignant tumor, is not suitable for a patient with an unstable condition, such as severe heart failure, whereas an examination of HA concentration only requires thoracentesis. In this case, the HA level was high, while the patient had neither malignant mesothelioma nor another pleural inflammatory disease. This may be attributed to MA infiltration of the pleura. To date, there has been no report of high HA levels in patients with MA; we believe that malignant cardiac tumors should be suspected in cases of a high HA concentration with heart failure.

We report a case of MA mimicking constrictive pericarditis, aortic dissection, and coronary artery disease. Careful assessment is necessary to determine the surgical strategy for MA. HA can be a helpful indicator for MA diagnosis. We believe that a more precise diagnosis can provide a more meaningful life for patients with MA.

## Supplementary Information

**Additional file 1: **Video 1

**Additional file 2: **Video 2

**Additional file 3: **Video 3

## Data Availability

All data generated or analyzed during this study are included in this published article and its supplementary information files.

## Abbreviations

MA: Mediastinal angiosarcoma
HA: Hyaluronic acid
MRI: Magnetic resonance imaging
POD: Postoperative day
